# Molecular docking analysis of a virulence factor protein dentilisin from *Treponema denticola* with oxazole piperazine derivatives

**DOI:** 10.6026/97320630019057

**Published:** 2023-01-31

**Authors:** Kandeeban Parthiban, Vishnu Priya Veeraraghavan, Surya Sekaran, Gayathri Rengasamy, Rajalakshmanan Eswaramoorthy

**Affiliations:** 1 Department of Biochemistry, Saveetha Dental College and Hospitals, Saveetha Institute of Medical and Technical Sciences, Saveetha University, Chennai-600077; 2 Department of Biomaterials (Green lab), Saveetha Dental College and Hospital, Saveetha Institute of Medical and Technical Sciences (SIMATS), Saveetha University, Chennai-600077

**Keywords:** antimicrobial agents, oxazole piperazine derivatives, *Treponema denticola*, Homology modeling, dentilisin

## Abstract

Dentilisin is a surface protease synthesized by the cell wall of *Treponema denticola*. This protein aids in the invasion of the periodontal tissue by causing infection. To identify drug molecules that have better results, homology modeling of the dentilisin protein was constructed, and molecular docking was performed with the oxazole compounds (1-6) taken from previous studies that are not yet clinically used. Data shows that compounds 1, 2, 3 show better inhibiting properties.

## Background:

*Treponema denticola* is a helical-shaped, oral spirochete, periodontal pathogen that inflicts damage on host tissue in conjunction with other members of an intricate polymicrobial oral biofilm. These members primarily reside in the subgingival and periodontal pockets, which increases gingival apoptotic cell death and results in hemolysis of human RBC [1,2]. In animal models, it can induce soft tissue degradation and loss of alveolar bone, and its levels rise in the subgingival microbiome of individuals with periodontal disease when compared to healthy people. An operon comprising the genes prcB, prcA, and prtP encodes dentilisin [3, 4, and 5]. Among these, prtP encodes for a 72 kDa protease domain that disintegrates extracellular matrix elements like fibronectin, activates C3, and contributes in fibrinogen attachment [6,7]. Additionally, dentilisin functions in immunomodulation, adhesion to fibronectin, as well as other processes. An active protease complex is synthesized on the surface of T. denticola by post-translational modification and interactions between both the expressed proteins [3,4,5, 6,7,8]. Dentilisin, also known as acylated chymotrypsin-like protease complex (CTLP), is a substantial virulence factor that triggers a spectrum of cytopathic effects that are consistent with the pathophysiology of periodontal disease [9,10]. An oxygen-containing heterocyclic molecule called oxazole is crucial in the development of several biologically efficient drug molecule, [12,13] including medications that are anti-inflammatory, anti-cancer, anti-depressant, anti-microbial, anti-obesity, anti-diabetic, and analgesic. Similarly, for several years, studies into the anti-microbial characteristics of pyrrole derivatives have driven the synthesis and analysis of a variety of novel compounds, including monodeoxypyoluteorin and derivatives of 2-(2'-hydroxybenzoyl) pyrrole bromine [14,15]. Therefore, it is of interest to document the molecular docking analysis data of a virulence factor protein dentilisin from Treponema denticola with oxazole piperazine derivatives for consideration in drug discovery.

## Material and Methods:

## Preparation of ligands:

**Figure 1:**
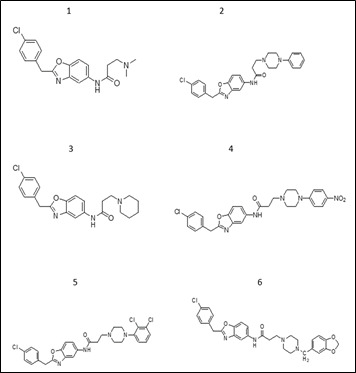
2D structures of the oxazole piperazine compounds (1-6)

The 2D mol structures of the selected oxazole piperazine derivatives (1-6) were drawn, and energy minimized using ChemDraw (Figure 1) and Chem3D software respectively (ChemOffice suite 16.0). During the optimization method, all parameters were selected in order to achieve a stable structure with the least amount of energy. Each molecule's 3D coordinates (PDB) were determined using optimized structure [16].

## Homology modeling of the dentilisin protein:

The fasta sequence of the protein dentilisin was derived from the Uniprot database (Uniprot ID: P96091). The query sequence is then submitted to the Swiss Model online server and the structure is built using the template with maximum sequence identity. Based on the structure assessment and Ramachandran favored region, the built model was further analyzed for protein preparation [17,18,19].

## Preparation of protein macromolecule:

**Figure 2:**
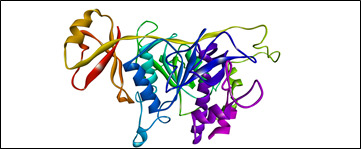
Homology modeled and prepared Dentilisin protein of *Treponema denticola*

The homology modeled structure (Figure 2) of the protein dentilisin of *Treponema denticola*, was prepared as per standard protocol practiced globally using the software Biovia Discovery tudio. Water molecules, other hetero atoms were chosen for elimination. The previously connected ligands were removed, and the protein was prepared by adding polar hydrogens and kollman charges with Auto Prep.

## Auto dock Vina analysis:

**Figure 3:**
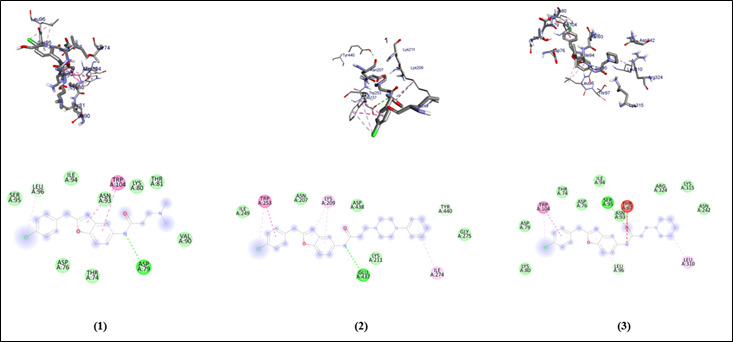
Molecular docking interaction analysis of compounds 1, 2, and 3 against the protein denilisin

**Figure 4:**
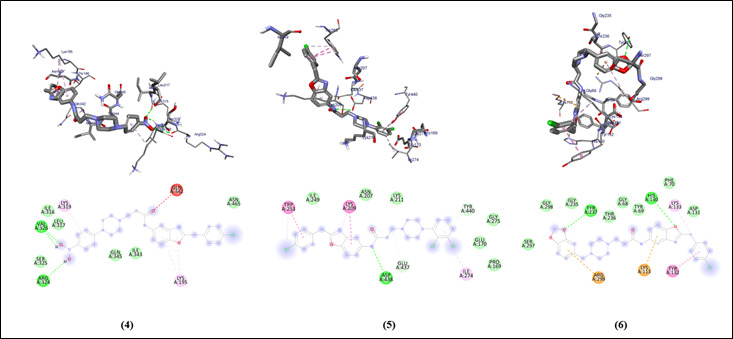
Molecular docking interaction analysis of compounds 4, 5, and 6 against the protein denilisin

The graphical user interface Auto Dock vina was used for Ligand-Protein docking interactions (Figure 3 and Figure 4). Auto Dock Tools (ADT), a free visual user interface (GUI) for the AutoDock Vina software, was used for the molecular docking research. For each ligand, nine alternative conformations were created and ranked based on their binding energies utilizing Auto Dock Vina algorithms.

## Drug-likeness and toxicity predictions: 

SwissADME and PROTOX-II online servers were used. This prediction points users in the direction of drug efficiency, gives information on whether or not the examined ligands have features consistent with becoming an orally active medication. This prediction is based on Lipinski et al's previously established idea known as Lipinski's rule of five [20].

## Statistical Analysis:

One way ANOVA was used for statistical analysis. The clinically proven drugs are used as control and the results are compared. The significance of the results was found to be p < 0.05.

## Results:

## Molecular docking interaction against protein dentilisin:

All compounds with the protein have binding energy in the range of -6.9 to -8.5 Kcal/mol (Table 1). The compounds show an H binding interaction similar to that of sulfamethoxazole (-5.6). Clinically proven drugs shown in lead binds to the binding site of protein. Azithromycin binds to the Gln-145, Glu-441, Thr-442, Lys-430 binding site. Sulfanilamide binds to the Asp-79, Asp-76, Thr-74 binding sites. Sulfamethoxazole binds to the Ser-291, Arg-299 binding site of the protein. All compounds show similar binding affinity as the lead molecules within the binding site.

## ADME and Lipinski rule of five:

The chosen compounds show a logKp value within the range of -6 to -5.12 cm/surface (Table 2). It is inferred that, more the negative value, the higher will be the permeation of skin. All the compounds show lesser negative values of log Kp than the lead compounds. The GI absorption of the compounds is high and there will be no involvement of carrier molecules to perform their function. This is similar to the control compound Sulfamethoxazole and Sulfanilamide. The compounds 4, 5 ,6 and the control group do not show any blood brain barrier permeability. However, compounds 1, 2, and 3 shows blood brain barrier permeation. The compound 4, 5, 6 and the control group taken doses not inhibit the CYP. Nonetheless, compounds 1, 2, and 3 are the inhibitors for CYP. Therefore, the compounds 1, 2, and 3 can be used as potentially lead drug molecules. The compounds 1, 2, and 3 obey the Lipinski rule of five and are similar to the control groups Sulfamethoxazole and Sulfanilamide (Table 3). Additionally, the control group molecule Azithromycin and compounds 4, 5, and 6 shows violation in the molecular weight (748.9, 519.98, 543.87, 533.02 respectively). Also, Azithromycin and compound 4 violates TPSA score (180.08, 107.43). In spite of these exceptions, this can still be used as a drug as these violations don't directly affect its function. Compared to this, the oxazole compounds don't have any violations and therefore can be used as lead molecules.

## Toxicity profile:

The compounds show class 4 toxicity and have inactive Immunotoxicity, cytotoxicity and hepatotoxicity (Table 4). The lethal dose parameters of the chosen molecules are lesser than the control group and thus can be used as drugs.

## Discussion:

The selected compounds (Oxazole piperazine derivatives) 5 (-8.5 kcal/mol) and 6(-8.4 kcal/mol) show better interaction compared to the clinically proven drugs (-6.9 kcal/mol & -7.9 kcal/mol). The compounds 1, 2, 3 obey Lipinski's rule of 5 similar to sulfanilamide and Sulfamethoxazole. Compounds 4, 5, 6 do not obey Lipinski's rule of 5 which is similar to Azithromycin. The selected compounds show better skin permeability (log Kp -6.28 to -5.12 (cm/s)). Compounds 1, 2, 3 & 5 have no toxicity. The reported compounds have stable H bonding, weak hydrophobic and van der Waals interactions within the binding pocket in targeted protein. The SwissADME prediction parameters showed that all the compounds (1- 6) have high gastrointestinal (GI) absorption, blood–brain barrier (BBB) permeation and all compounds are substrates of permeability glycoprotein (P-gp) except 1. The CYP's interaction result showed that all the compounds are inhibitors for CYP2C19, CYP2C9, and CYP2D6. Compounds 4, 5 and 6 were found to be potential inhibitors for CYP1A2. For CYP3A4, compound Azithromycin, sulfanilamide and sulfamethoxazole were found to be non-inhibitors whereas compounds 1-6 were found to be potential inhibitors [21].

## Conclusion:

The selected ligands (1-6) show optimal interactions with modeled protein within the binding sites. Ligands 1, 2, and 3 obeys Lipinski's rule of 5 with low toxicity profile and have better interaction score (-6.9, -7.9, and -7.5 kcal/mol) than Sulfanilamide and Sulfamethoxazole (-5.6 and -6.2 kcal/mol). Therefore, compounds 1, 2 & 3 are inferred to be the potential inhibitors for the virulence factor dentilisin.

## Figures and Tables

**Table 1:** Molecular docking scores and residual amino acid interactions of oxazole compounds (1-6) along with control groups against Dentilisin of *Treponema denticola* (Homology modeled).

Ligands	Docking scores/Affinity (kcal/mol)	H-bond	Amino Acid Residual interactions	
			Hydrophobic/Pi-Cation	Van dar Waals
1	-6.9	Asp-79	Leu-96, Trp-104	Ser:95, Ile-94, Asn-93, Lys-80, Thr-81, Val-90, Thr-74, Asp-76
2	-7.9	Glu-437	Trp-253, Lys-209, Ile-274, Tyr-440	Ile-249, Asn-207, Asp-438, Lys-211, Gly-275
3		Ser-95	Trp-104, Thr-97, Leu-96, Leu-310	Lys-80, Asp-79, Thr-74, Asp-76, Ile-94, Asn-93, Arg-324, Lys-315, Asn-242
	-7.5			
4	-7.8	Val-326, Arg324	Lys-319, Gln-342, Lys-195	Ser-325, Ile-316, Leu-317, Gln-345, Ile-343, Asn-465
5	-8.5	Asp-438	Trp-253, Lys-209, Glu-437, Try-440, Ile-274	Ile-249, Asn-207, Lys-211, Gly-275, Glu-170, Pro-169
6	-8.4	Try-237, His-130	Arg-299, Lys-113, Tyr-132, Lys-133	Ser-297, Gly-298, Gly-235, Thr-236, Gly-68, Thr-69, Phe-70, Asp-131
Azithromycin	-6.7	Gln-145, Glu-441, Thr-442, Lys-430	Pro-169, Asn-144	Ser-153, Pro-152, Asp-151, Thr-440, Asn-173
Sulfanilamide	-5.6	Asp-79, Asp-76, Thr-74	Trp-104	Thr-102, Ile-94, Tyr-103, Asn-93
Sulfamethoxazole	-6.2	Ser-291, Arg-299	Ser-302, Tyr-237	Ile-238, Tyr-224, Trp-301, Leu-223, Gly-293, Gly-298, Ser-297, Glu-295, Lys-133,

**Table 2:** ADMET and SwissADME properties of oxazole compounds (1-6)

Compound	log Kp (cm/s)	GI absorption	BBB permeant	Pgp substrate	CYP1A2 inhibitor	CYP2C19 inhibitor	CYP2C9 inhibitor	CYP2D6 inhibitor	CYP3A4 inhibitor
1	-6	High	Yes	No	Yes	Yes	Yes	Yes	Yes
2	-5.58	High	Yes	Yes	Yes	Yes	Yes	Yes	Yes
3	-5.65	High	Yes	Yes	Yes	Yes	Yes	Yes	Yes
4	-5.98	High	No	Yes	No	Yes	Yes	Yes	Yes
5	-5.12	High	No	Yes	No	Yes	Yes	Yes	Yes
6	-6.28	High	No	Yes	No	Yes	Yes	Yes	Yes
Azithromycin	-8.01	Low	No	Yes	No	No	No	No	No
Sulfanilamide	-7.79	High	No	No	No	No	No	No	No
Sulfamethoxazole	-7.21	High	No	No	No	No	No	No	No

**Table 3:** Lipinski & Veber rule.

Compound	MW	iLogP	HBD (nOHNH)	HBA (nON)	nrotb	MR	TPSA	Lipinski #violations	Bio availability score
Lipinski*	≤500	≤5	≤5	≤10	≤10	-	-		
Veber**	-	-	-	-	-	-	≤ 140		
1	357.83	3.46	1	4	7	100.1	58.37	0	0.55
2	474.98	4.31	1	4	8	143.37	61.61	0	0.55
3	397.9	3.97	1	4	7	116.32	58.37	0	0.55
4	519.98	3.32	1	6	9	152.19	107.43	1	0.55
5	543.87	4.42	1	4	8	153.39	61.61	2	0.17
6	533.02	4.93	1	7	9	153.68	80.07	1	0.55
Azithromycin	748.98	4.76	5	14	7	200.78	180.08	2	0.17
Sulfanilamide	172.2	0.61	2	3	1	41.84	94.56	0	0.55
Sulfamethoxazole	253.28	1.03	2	4	3	62.99	106.6	0	0.55

**Table 4:** Toxicity profile of the oxazole compounds (1-6)

			Toxicity				
Compound	aLD50 (mg/kg)	Class	HEPATOTOXICITY	CARCINOGENICITY	IMMUNOTOXICITY	MUTAGENICITY	CYTOTOXICITY
1	1000mg/kg	4	Inactive	Inactive	Inactive	Inactive	Inactive
2	1600mg/kg	4	Inactive	Inactive	Inactive	Inactive	Inactive
3	1500mg/kg	4	Inactive	Inactive	Inactive	Inactive	Inactive
4	1420mg/kg	4	Inactive	Active	Inactive	Active	Inactive
5	1500mg/kg	4	Inactive	Inactive	Inactive	Inactive	Inactive
6	1000mg/kg	4	Inactive	Active	Inactive	Active	Inactive
Azithromycin	2000mg/kg	4	Inactive	Inactive	Active	Inactive	Inactive
Sulfanilamide	3000mg/kg	5	Inactive	Active	Inactive	Inactive	Inactive
Sulfamethoxazole	2300mg/kg	5	Active	Active	Inactive	Inactive	Inactive
^a^ LD_50_: lethal dose parameter
